# Certain Cases of Painful Menstruation

**Published:** 1895-05-25

**Authors:** George Drummond Robinson

**Affiliations:** Obstetric Physician to the St. George's and St. James' Dispensary, and Physician to Out-Patients, British Lying-In Hospital


### CERTAIN CASES OF PAINFUL MENSTRUATION.

By George Drummond Robinson, M.D. and B.S. Lond., M.R.C.P., Obstetric Physician to the St. George's and St. James' Dispensary, and Physician to Out-Patients, British Lying-In Hospital.

The high standard of civilisation attained in our day has been by no means an unmixed blessing to the human race. With its enormous advantages have come always attendant evils. Its injurious influences are to be traced in every branch of pathology. Here we find them startlingly obvious, thrusting themselves on the notice of the most inattentive observer, there indistinctly visible amidst the thousand forces of heredity, acting on us through a long line of forefathers, or almost indistinguishably mingled with the other complex factors of our daily environment. But always there, obvious or hidden, potent or feeble, working us evil, ceaseless, relentless, ubiquitous.

Perhaps in no part of the domain of pathology is this fact more apparent than in that dealing with the nervous system. To take one morbid symptom alone connected with this branch of the science—pain. There seems no doubt that the higher the state of civilisation the commoner and more marked is pain. Processes entirely physiological in our earlier ancestors or in peoples low down in the scale of civilisation are becoming with us daily more and more pathological by reason of their complication with this symptom. Of this, menstruation is a striking example.

Whilst amongst savage peoples menstruation is, in the vast majority of cases, a purely physiological function, as seldom accompanied with pain as is the process of digestion, with us few women pass through menstrual life without at some time or other suffering pain of greater or less intensity at the menstrual periods.

The pain accompanying menstruation may be situated anywhere in the body and may be of any degree of severity. In its most marked forms it originates in the pelvic sexual organs and is situated in the lower part of the abdomen or back, from which regions it may radiate in various directions. In many instances this pelvic pain is associated with morbid anatomical conditions of the pelvic organs, such as maldevelopment of the uterus and its appendages, exfoliation of the uterine mucous membrane, uterine new growths, and the various pelvic inflammations, acute or chronic.

In a very large number of cases, many of them of the most severe type, the pelvic organs appear to be absolutely normal, and the sufferers are in every other respect perfectly healthy. It is to this latter class of cases, known by such names as spasmodic, neuralgic, or obstructive dysmenorrhœa, that I would direct attention.

The pain may first show itself at any period of life; most frequently it comes on at puberty, but to this there are many exceptions. It is experienced in the lower part of the back or abdomen, and from thence perhaps shoots down the thighs. Paroxysmal in character it comes on in pangs which last a longer or shorter time, and are separated by intervals of comparative freedom from pain. In these respects the pain is very similar to that of labour. During one of these paroxysms, in a severe case, the patient presents all the appearances that accompany the severest forms of abdominal pain, such as that due to biliary, renal, or intestinal colic. Unconscious of all save her own terrible distress, with stiffened limbs and breath long held, the sufferer lies wherever she may have fallen, writhing and groaning in agony. The skin is pale and covered with a cold sweat, there is retching and vomiting, the pulse is small and rapid, occasionally there is maniacal excitement, such as is sometimes seen during labour. On the other extreme, in the mildest cases, the pain is so slight as to be little more than discomfort.

As a rule, these attacks precede the menstrual flow by some hours the pain continues after the flow appears, and gradually subsides as this becomes well established. In most of these cases the menstrual loss is scanty. Should a patient suffering in this way become pregnant, a labour at or near the full time will probably completely cure or, at any rate, greatly relieve the condition. Experience, however, teaches us that such persons are apt to remain sterile, and, if that should be the case, marriage almost invariably aggravates the pain. One frequently meets with cases of women who, having for years endured the pain of this form of dysmenorrhœa without medical advice, are driven to seek relief to their intolerable sufferings after a few months of sterile married life.

Passing completely over the vexed question of the pathology of this condition, it will be useful to regard the subject entirely from the point of view of treatment, and to consider the best methods of relieving such cases.

Hygienic measures—so often underrated or entirely overlooked—are of the very greatest importance. There should be exercise and rest both for mind and body, carefully regulated according to the requirements of the individual case. Any morbid condition, such as anæmia or, most important of all, constipation, should be appropriately treated.

The menstrual loss, usually scanty, may often with benefit be encouraged by the old-fashioned and popular method of combining a hot foot-bath with a dose of diffusible stimulant.

Of drugs directly attacking the pain I have found a mixture of bromide of potassium with antipyrin given in small doses, repeated at frequent intervals during the attack, most useful. I have also found the tincture of castoreum, phenacetin, and exalgine successful in some cases. Other drugs as guaiacum, nitroglycerine, and cannabis indica are worthy of a trial. Opium or morphia should never be given, for since the pain recurs month after month for many years the opium or morphia habit is exceedingly liable to be established. In a certain number of the most distressing cases drugs have little or no effect in relieving the pain. Under such circumstances the most brilliant results may follow operative treatment. That operative treatment is unsuitable for all cases and should be resorted to only in a limited number cannot be too strongly insisted upon. If after a thorough trial drugs fail and the pain is so severe as to affect the general health or interfere seriously with the performance of the patient's duties, then operative treatment holds out a fair prospect of cure or at any rate of considerable relief.

Of the many operations practised for the relief of this morbid condition, I can from personal experience strongly recommend rapid dilatation of the internal os uteri by means of graduated bougies. In my experience it is quite as effective as the other procedures, and if carefully performed is practically free from any danger to the patient. An anæsthetic should always be administered. In my student days I have seen this operation performed without anæsthesia. The region of the internal os (and to a less degree the endometrium also) is, however, very sensitive in these cases, and the passage of the bougie invariably sets up the characteristic pain with such intensity that it is impossible to dilate it thoroughly at a single operation without anæsthesia.

An antiseptic douche is first given, and the anterior lip of the cervix uteri being seized with Volsella forceps, traction is made until the external os presents at the vaginal orifice. Graduated bougies (Hegar's answer admirably) are now passed one after the other until by the feeling of resistance experienced at the internal os, it is judged that that orifice has been thoroughly stretched. To dilate beyond this degree is unnecessary, and not devoid of danger.

After the operation the patient is kept in bed for a day or two. The insertion of an intra-uterine stern pessary after dilatation is of very doubtful value, and exposes the patient to risk of pelvic inflammation.

In the majority of cases this simple operation relieves the pain—sometimes permanently, sometimes only for a time. Should the pain return again after some months' absence the operation may be repeated, and if carefully performed is quite free from serious danger.

In many cases of sterile women cure of the pain by dilatation is rapidly followed by pregnancy. As before mentioned, the fact that all cases of painful menstruation are not suitable for this method of treatment must ever be borne in mind. There are many varieties of painful menstruation, and for treatment by dilatation the cases must be carefully selected.

